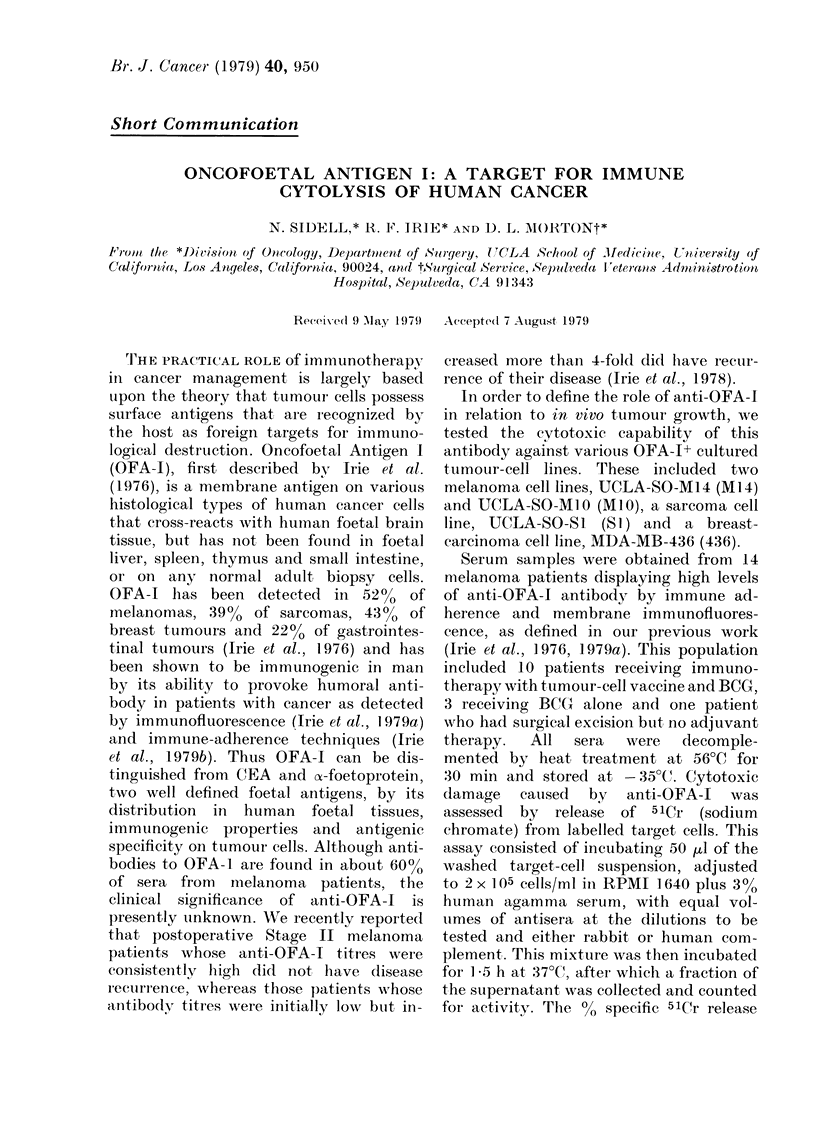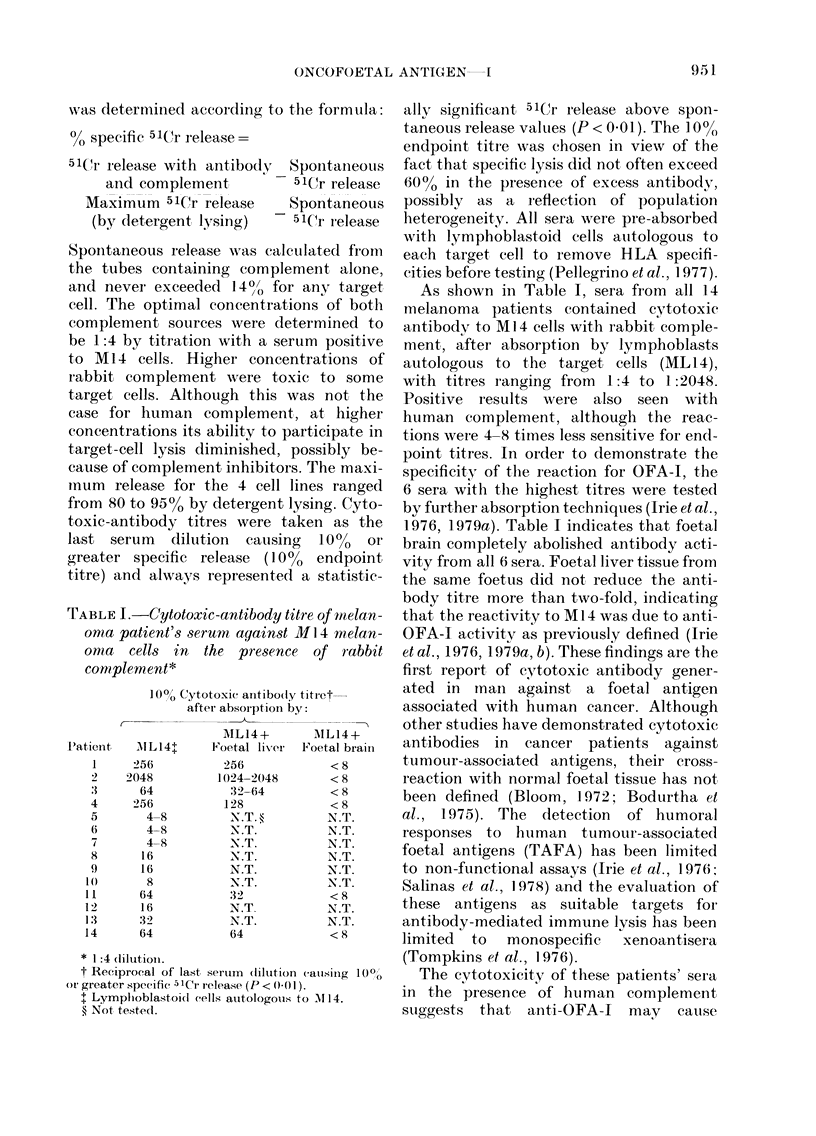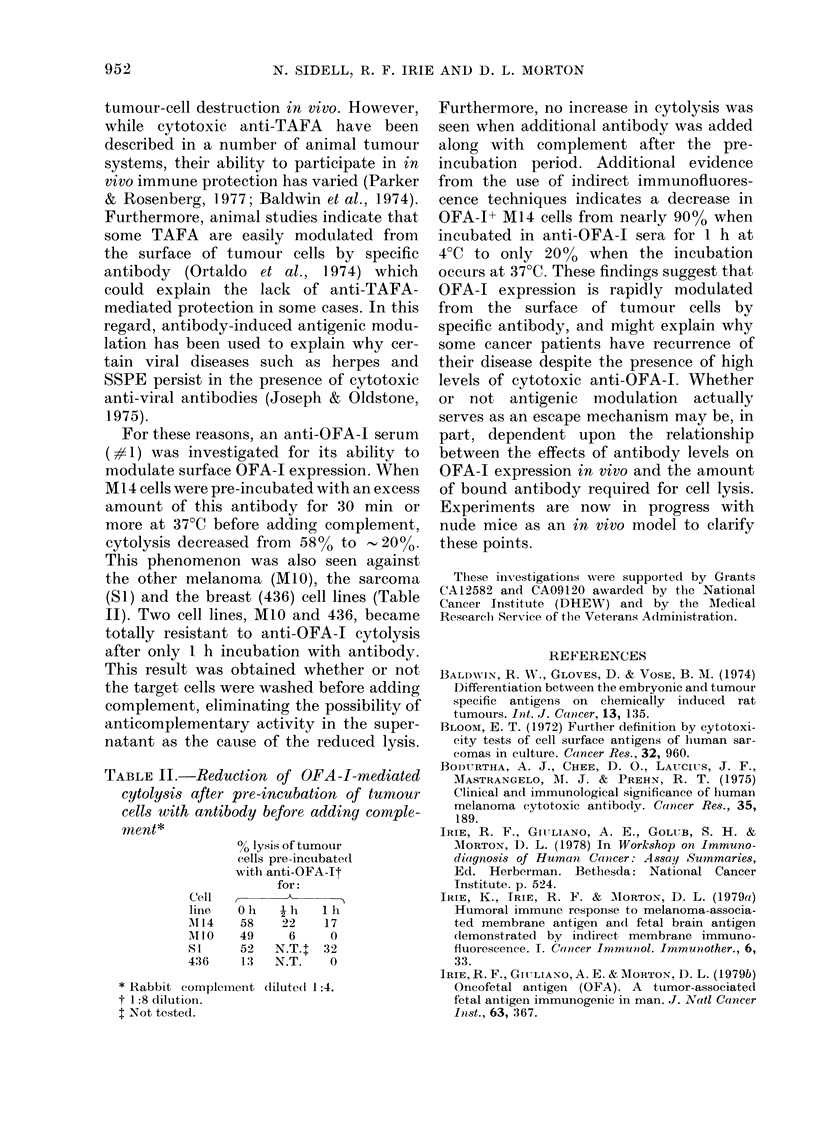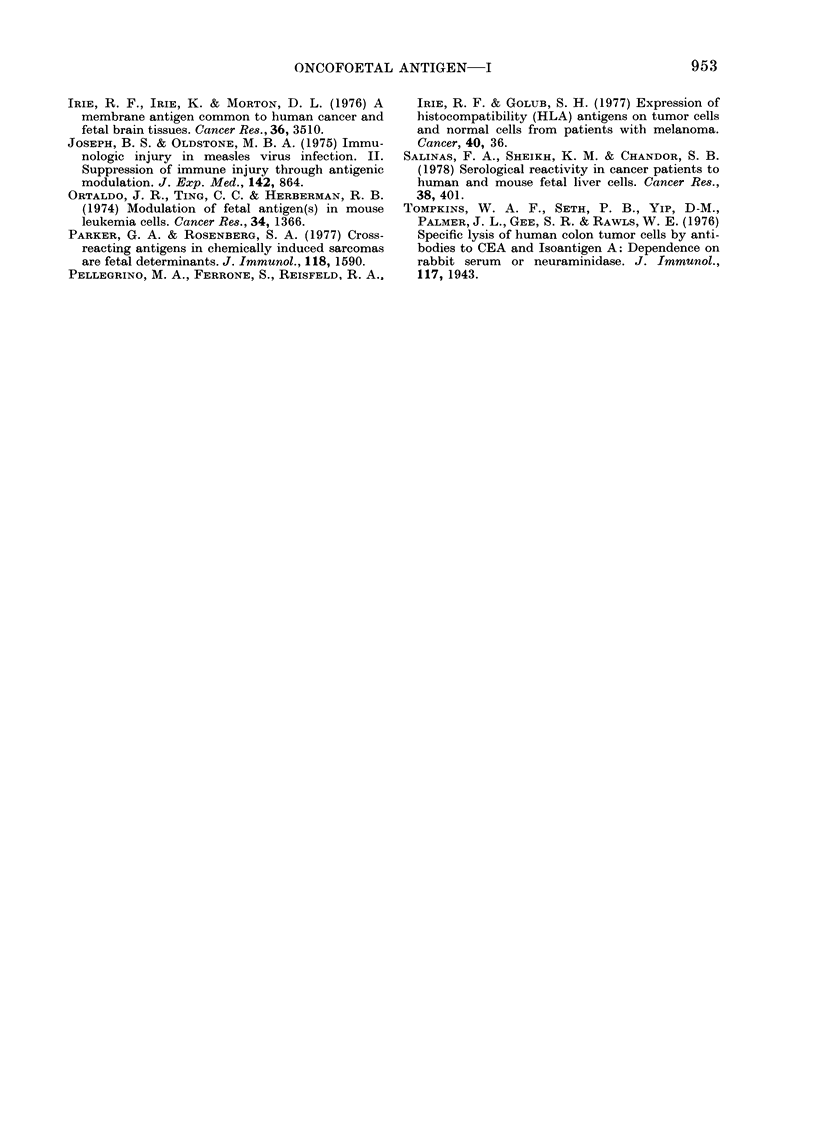# Oncofoetal antigen I: a target for immune cytolysis of human cancer.

**DOI:** 10.1038/bjc.1979.291

**Published:** 1979-12

**Authors:** N. Sidell, R. F. Irie, D. L. Morton


*Br. J. Cancer* (1979) **40**, 950

***Short Communication***

### ONCOFOETAL ANTIGEN I: A TARGET FOR IMMUNE CYTOLYSIS OF HUMAN CANCER

N. SIDELL,* R. F. IRIE* AND D. L. MORTON†*

*From the *Division of Oncology, Department of Surgery, UCLA School of Medicine, University of California, Los Angeles, California, 90024, and †Surgical Service, Sepulveda Veterans Administrotion Hospital, Sepulveda, CA 91343*

Received 9 May 1979 Accepted 7 August 1979

THE PRACTICAL ROLE of immunotherapy in cancer management is largely based upon the theory that tumour cells possess surface antigens that are recognized by the host as foreign targets for immunological destruction. Oncofoetal Antigen I (OFA-I), first described by Irie *et al.* (1976), is a membrane antigen on various histological types of human cancer cells that cross-reacts with human foetal brain tissue, but has not been found in foetal liver, spleen, thymus and small intestine, or on any normal adult biopsy cells. OFA-I has been detected in 52% of melanomas, 39% of sarcomas, 43% of breast tumours and 22% of gastrointestinal tumours (Irie *et al.*, 1976) and has been shown to be immunogenic in man by its ability to provoke humoral antibody in patients with cancer as detected by immunofluorescence (Irie *et al.*, 1979*a*) and immune-adherence techniques (Irie *et al.*, 1979*b*). Thus OFA-I can be distinguished from CEA and $\alpha$-foetoprotein, two well defined foetal antigens, by its distribution in human foetal tissues, immunogenic properties and antigenic specificity on tumour cells. Although antibodies to OFA-I are found in about 60% of sera from melanoma patients, the clinical significance of anti-OFA-I is presently unknown. We recently reported that postoperative Stage II melanoma patients whose anti-OFA-I titres were consistently high did not have disease recurrence, whereas those patients whose antibody titres were initially low but increased more than 4-fold did have recurrence of their disease (Irie *et al.*, 1978).

In order to define the role of anti-OFA-I in relation to *in vivo* tumour growth, we tested the cytotoxic capability of this antibody against various OFA-I$^+$ cultured tumour-cell lines. These included two melanoma cell lines, UCLA-SO-M14 (M14) and UCLA-SO-M10 (M10), a sarcoma cell line, UCLA-SO-S1 (S1) and a breast-carcinoma cell line, MDA-MB-436 (436).

Serum samples were obtained from 14 melanoma patients displaying high levels of anti-OFA-I antibody by immune adherence and membrane immunofluorescence, as defined in our previous work (Irie *et al.*, 1976, 1979*a*). This population included 10 patients receiving immunotherapy with tumour-cell vaccine and BCG, 3 receiving BCG alone and one patient who had surgical excision but no adjuvant therapy. All sera were decomplemented by heat treatment at 56°C for 30 min and stored at −35°C. Cytotoxic damage caused by anti-OFA-I was assessed by release of $^{51}Cr$ (sodium chromate) from labelled target cells. This assay consisted of incubating 50 $\mu$l of the washed target-cell suspension, adjusted to $2 \times 10^5$ cells/ml in RPMI 1640 plus 3% human agamma serum, with equal volumes of antisera at the dilutions to be tested and either rabbit or human complement. This mixture was then incubated for 1·5 h at 37°C, after which a fraction of the supernatant was collected and counted for activity. The % specific $^{51}Cr$ release was determined according to the formula:

$$\% \text{ specific } ^{51}\text{Cr release} = \frac{^{51}\text{Cr release with antibody and complement} - \text{Spontaneous } ^{51}\text{Cr release}}{\text{Maximum } ^{51}\text{Cr release (by detergent lysing)} - \text{Spontaneous } ^{51}\text{Cr release}}$$

Spontaneous release was calculated from the tubes containing complement alone, and never exceeded 14% for any target cell. The optimal concentrations of both complement sources were determined to be 1:4 by titration with a serum positive to M14 cells. Higher concentrations of rabbit complement were toxic to some target cells. Although this was not the case for human complement, at higher concentrations its ability to participate in target-cell lysis diminished, possibly because of complement inhibitors. The maximum release for the 4 cell lines ranged from 80 to 95% by detergent lysing. Cytotoxic-antibody titres were taken as the last serum dilution causing 10% or greater specific release (10% endpoint titre) and always represented a statistically significant $^{51}$Cr release above spontaneous release values ($P < 0{\cdot}01$). The 10% endpoint titre was chosen in view of the fact that specific lysis did not often exceed 60% in the presence of excess antibody, possibly as a reflection of population heterogeneity. All sera were pre-absorbed with lymphoblastoid cells autologous to each target cell to remove HLA specificities before testing (Pellegrino *et al.*, 1977).

TABLE I.—*Cytotoxic-antibody titre of melanoma patient's serum against M*14 *melanoma cells in the presence of rabbit complement**

| | 10% Cytotoxic antibody titre†—after absorption by: | | |
|---|---|---|---|
| Patient | ML14‡ | ML14+ Foetal liver | ML14+ Foetal brain |
| 1 | 256 | 256 | < 8 |
| 2 | 2048 | 1024–2048 | < 8 |
| 3 | 64 | 32–64 | < 8 |
| 4 | 256 | 128 | < 8 |
| 5 | 4–8 | N.T.§ | N.T. |
| 6 | 4–8 | N.T. | N.T. |
| 7 | 4–8 | N.T. | N.T. |
| 8 | 16 | N.T. | N.T. |
| 9 | 16 | N.T. | N.T. |
| 10 | 8 | N.T. | N.T. |
| 11 | 64 | 32 | < 8 |
| 12 | 16 | N.T. | N.T. |
| 13 | 32 | N.T. | N.T. |
| 14 | 64 | 64 | < 8 |

* 1:4 dilution.

† Reciprocal of last serum dilution causing 10% or greater specific $^{51}$Cr release ($P < 0{\cdot}01$).

‡ Lymphoblastoid cells autologous to M14.

§ Not tested.

As shown in Table I, sera from all 14 melanoma patients contained cytotoxic antibody to M14 cells with rabbit complement, after absorption by lymphoblasts autologous to the target cells (ML14), with titres ranging from 1:4 to 1:2048. Positive results were also seen with human complement, although the reactions were 4–8 times less sensitive for endpoint titres. In order to demonstrate the specificity of the reaction for OFA-I, the 6 sera with the highest titres were tested by further absorption techniques (Irie *et al.*, 1976, 1979*a*). Table I indicates that foetal brain completely abolished antibody activity from all 6 sera. Foetal liver tissue from the same foetus did not reduce the antibody titre more than two-fold, indicating that the reactivity to M14 was due to anti-OFA-I activity as previously defined (Irie *et al.*, 1976, 1979*a*, *b*). These findings are the first report of cytotoxic antibody generated in man against a foetal antigen associated with human cancer. Although other studies have demonstrated cytotoxic antibodies in cancer patients against tumour-associated antigens, their cross-reaction with normal foetal tissue has not been defined (Bloom, 1972; Bodurtha *et al.*, 1975). The detection of humoral responses to human tumour-associated foetal antigens (TAFA) has been limited to non-functional assays (Irie *et al.*, 1976; Salinas *et al.*, 1978) and the evaluation of these antigens as suitable targets for antibody-mediated immune lysis has been limited to monospecific xenoantisera (Tompkins *et al.*, 1976).

The cytotoxicity of these patients' sera in the presence of human complement suggests that anti-OFA-I may cause tumour-cell destruction *in vivo*. However, while cytotoxic anti-TAFA have been described in a number of animal tumour systems, their ability to participate in *in vivo* immune protection has varied (Parker & Rosenberg, 1977; Baldwin *et al*., 1974). Furthermore, animal studies indicate that some TAFA are easily modulated from the surface of tumour cells by specific antibody (Ortaldo *et al*., 1974) which could explain the lack of anti-TAFA-mediated protection in some cases. In this regard, antibody-induced antigenic modulation has been used to explain why certain viral diseases such as herpes and SSPE persist in the presence of cytotoxic anti-viral antibodies (Joseph & Oldstone, 1975).

For these reasons, an anti-OFA-I serum (#1) was investigated for its ability to modulate surface OFA-I expression. When M14 cells were pre-incubated with an excess amount of this antibody for 30 min or more at 37°C before adding complement, cytolysis decreased from 58% to ~20%. This phenomenon was also seen against the other melanoma (M10), the sarcoma (S1) and the breast (436) cell lines (Table II). Two cell lines, M10 and 436, became totally resistant to anti-OFA-I cytolysis after only 1 h incubation with antibody. This result was obtained whether or not the target cells were washed before adding complement, eliminating the possibility of anticomplementary activity in the supernatant as the cause of the reduced lysis.

TABLE II.—*Reduction of OFA-I-mediated cytolysis after pre-incubation of tumour cells with antibody before adding complement**

| Cell line | % lysis of tumour cells pre-incubated with anti-OFA-I† for: 0 h | ½ h | 1 h |
|---|---|---|---|
| M14 | 58 | 22 | 17 |
| M10 | 49 | 6 | 0 |
| S1 | 52 | N.T.‡ | 32 |
| 436 | 13 | N.T. | 0 |

* Rabbit complement diluted 1:4.

† 1:8 dilution.

‡ Not tested.

Furthermore, no increase in cytolysis was seen when additional antibody was added along with complement after the pre-incubation period. Additional evidence from the use of indirect immunofluorescence techniques indicates a decrease in OFA-I+ M14 cells from nearly 90% when incubated in anti-OFA-I sera for 1 h at 4°C to only 20% when the incubation occurs at 37°C. These findings suggest that OFA-I expression is rapidly modulated from the surface of tumour cells by specific antibody, and might explain why some cancer patients have recurrence of their disease despite the presence of high levels of cytotoxic anti-OFA-I. Whether or not antigenic modulation actually serves as an escape mechanism may be, in part, dependent upon the relationship between the effects of antibody levels on OFA-I expression *in vivo* and the amount of bound antibody required for cell lysis. Experiments are now in progress with nude mice as an *in vivo* model to clarify these points.

These investigations were supported by Grants CA12582 and CA09120 awarded by the National Cancer Institute (DHEW) and by the Medical Research Service of the Veterans Administration.

REFERENCES

BALDWIN, R. W., GLOVES, D. & VOSE, B. M. (1974) Differentiation between the embryonic and tumour specific antigens on chemically induced rat tumours. *Int. J. Cancer*, **13**, 135.

BLOOM, E. T. (1972) Further definition by cytotoxicity tests of cell surface antigens of human sarcomas in culture. *Cancer Res*., **32**, 960.

BODURTHA, A. J., CHEE, D. O., LAUCIUS, J. F., MASTRANGELO, M. J. & PREHN, R. T. (1975) Clinical and immunological significance of human melanoma cytotoxic antibody. *Cancer Res*., **35**, 189.

IRIE, R. F., GIULIANO, A. E., GOLUB, S. H. & MORTON, D. L. (1978) In *Workshop on Immunodiagnosis of Human Cancer: Assay Summaries*, Ed. Herberman. Bethesda: National Cancer Institute. p. 524.

IRIE, K., IRIE, R. F. & MORTON, D. L. (1979*a*) Humoral immune response to melanoma-associated membrane antigen and fetal brain antigen demonstrated by indirect membrane immunofluorescence. I. *Cancer Immunol. Immunother*., **6**, 33.

IRIE, R. F., GIULIANO, A. E. & MORTON, D. L. (1979*b*) Oncofetal antigen (OFA). A tumor-associated fetal antigen immunogenic in man. *J. Natl Cancer Inst*., **63**, 367.

IRIE, R. F., IRIE, K. & MORTON, D. L. (1976) A membrane antigen common to human cancer and fetal brain tissues. *Cancer Res.*, **36**, 3510.

JOSEPH, B. S. & OLDSTONE, M. B. A. (1975) Immunologic injury in measles virus infection. II. Suppression of immune injury through antigenic modulation. *J. Exp. Med.*, **142**, 864.

ORTALDO, J. R., TING, C. C. & HERBERMAN, R. B. (1974) Modulation of fetal antigen(s) in mouse leukemia cells. *Cancer Res.*, **34**, 1366.

PARKER, G. A. & ROSENBERG, S. A. (1977) Cross-reacting antigens in chemically induced sarcomas are fetal determinants. *J. Immunol.*, **118**, 1590.

PELLEGRINO, M. A., FERRONE, S., REISFELD, R. A., IRIE, R. F. & GOLUB, S. H. (1977) Expression of histocompatibility (HLA) antigens on tumor cells and normal cells from patients with melanoma. *Cancer*, **40**, 36.

SALINAS, F. A., SHEIKH, K. M. & CHANDOR, S. B. (1978) Serological reactivity in cancer patients to human and mouse fetal liver cells. *Cancer Res.*, **38**, 401.

TOMPKINS, W. A. F., SETH, P. B., YIP, D-M., PALMER, J. L., GEE, S. R. & RAWLS, W. E. (1976) Specific lysis of human colon tumor cells by antibodies to CEA and Isoantigen A: Dependence on rabbit serum or neuraminidase. *J. Immunol.*, **117**, 1943.